# 3’-hydroxypuerarin mitigates LPS-induced acute lung injury by inhibiting TLR4 activation-mediated NF-κB p65/NLRP3/GSDMD signaling

**DOI:** 10.3389/fimmu.2026.1701778

**Published:** 2026-03-31

**Authors:** Huiyu Hu, Kongyan Wang, Yi Qiu, Zaibin Xu, Yan Chen, Yingjie Hu, Jiawen Huang, Zhuohui Luo

**Affiliations:** 1 Hainan Pharmaceutical Research and Development Science Park, Hainan Medical University, Haikou, China; 2 Science and Technology Innovation Center, Guangzhou University of Chinese Medicine, Guangzhou, China

**Keywords:** 3’-hydroxypuerarin, acute lung injury, anti-inflammation, NF-κB p65 signaling, NLRP3/GSDMD signaling, TLR4 activation

## Abstract

**Introduction:**

Acute lung injury (ALI) is a common critical respiratory illness. 3’-Hydroxypuerarin (3HP), a beneficial isoflavone from *Pueraria lobata* (Willd.) Ohwi, possesses significant pharmacological activities, but its effects on ALI remain limited.

**Methods:**

To investigate the role of 3HP in mitigating ALI, *in vivo* lipopolysaccharide (LPS)- induced ALI in mice and *in vitro* LPS-induced RAW264.7 macrophage inflammatory injury were carried out using ELISA kits, RT-qPCR, immunofluorescence, Western blotting, molecular docking, and molecular dynamics simulation analyses.

**Results:**

3HP significantly reduced IL6 and TNF-α levels in bronchoalveolar lavage fluid (BALF) and serum, attenuated pulmonary edema, inhibited the mRNA levels of chemokines and inflammatory factors in lung tissues, and suppressed the expression of IL1β, IL6, TNF-α, HMGB1, TLR4, MyD88, p-IκBα (S32/S36), p-NF-κB p65 (S536), COX2, iNOS, ICAM1, VCAM1, NLRP3, ASC, Caspase-1, Cleaved Caspase-1 (Ala317) p10, NEK7, Caspase-8, IL18, GSDMD, and GSDMD N-terminal both in lung tissues and in RAW264.7 cells, indicating that 3HP inhibited LPS-stimulated TLR4 activation, thereby reducing IκBα phosphorylation and degradation, and preventing NF-κB p65 nuclear translocation to mediate the transcription and expression of inflammatory mediators. It also inhibited NLRP3 inflammasome activation, decreased Caspase-1 cleavage of GSDMD, and lowered the release of the pore-forming GSDMD N-terminal structural domain, blocking the immune response to pyroptosis. Moreover, the NLRP3 inhibitor MCC950 enhanced the effect of 3HP on acute cellular inflammatory injury. Notably, molecular docking and dynamics simulation revealed that 3HP stably bound to TLR4. Resatorvid (TAK-242), a selective TLR4 signaling inhibitor, significantly enhanced 3HP’s inhibitory effect on TLR4, further indicating that 3HP may block the NF-κB p65/NLRP3/GSDMD signaling pathway by inhibiting TLR4 activation in response to LPS stimulation.

**Conclusions:**

Our results demonstrated that 3HP mitigates LPS-induced ALI by inhibiting TLR4 activation-mediated NF-κB p65/NLRP3/GSDMD signaling. These findings provide scientific evidence for the clinical treatment of ALI and present new insights into the pharmacological role of 3HP in mitigating acute lung inflammatory diseases.

## 1 Introduction

Acute lung injury (ALI) is a severe respiratory disease caused by endogenous and exogenous pathogenic factors, clinically characterized by manifestation of acute hypoxic respiratory failure with bilateral pulmonary infiltrates and noncardiogenic hypoxemia, with a worldwide prevalence (1, 2). It mainly presents as an uncontrolled inflammatory response, oxidative stress, pulmonary edema, and the presence of inflammatory cells, eventually progressing to acute respiratory distress syndrome (ARDS) (3). Although mechanical ventilation effectively alleviates inflammatory lung injury, the mortality rate of ALI/ARDS remains high at 40 to 60% (4, 5). Therefore, clarifying ALI’s specific pathogenesis and pathophysiological mechanisms and exploring effective therapeutic options is crucial.

Macrophages, a vital part of the innate immune system, combat infections, eliminate inflammatory responses, and facilitate tissue repair (6, 7). Numerous studies have demonstrated that infection with lipopolysaccharide (LPS), a major component of the outer membrane of Gram-negative bacteria, triggers overactivation of macrophages, promoting lung inflammation and damage, thereby exacerbating the onset and progression of ALI (8). Consequently, LPS is widely used to model ALI/ARDS in rodents (9). Common induction routes include intratracheal instillation (typically 2 to 10 mg/kg), intraperitoneal injection (5 to 20 mg/kg), and intravenous injection (10). LPS stimulation triggers a series of intracellular signaling events, including binding to toll-like receptor 4 (TLR4), activating the myeloid differentiation primary response protein 88 (MyD88)-nuclear factor-kappa B (NF-κB) signaling pathway, and promoting the nuclear translocation of NF-κB. This process regulates the transcription of inflammatory mediators and the release of pro-inflammatory cytokines and chemokines, such as interleukin 6 (IL6), tumor necrosis factor-α (TNF-α), and interleukin 1β (IL1β) (11, 12). Additionally, LPS exposure triggers the assembly of the macrophage NOD-like receptor family pyrin domain containing 3 (NLRP3) inflammasome and activates a subsequent inflammatory cascade response (13, 14). Indeed, the activated downstream protease, Caspase-1, cleaves the substrate GSDMD, releasing its N-terminal domain and triggering membrane pore formation and the immune response associated with cellular pyroptosis (15). Furthermore, activated Caspase-1 cleaves the precursor inflammatory cytokines pro-IL1β and pro-IL18 to release mature IL1β and IL18 (16, 17), which are released through molecular pores in the cell membrane and expelled from the cell, thereby worsening the inflammatory cascade response and influencing the progression of ALI (18, 19). Thus, pharmacological inhibition of macrophage NF-κB signaling and NLRP3 inflammasome activation represents a viable approach to alleviate ALI.

Traditional Chinese medicine (TCM) has a rich history of effectively preventing and treating various illnesses with few side effects (20). Classical TCM strategies for ALI primarily focus on clearing heat-toxin, resolving phlegm, and relieving lung obstruction. They often use formulas such as Yupingfeng San (which regulates immunity and protects the lung barrier) and Gegentang (derived from Pueraria lobata and exerting anti-inflammatory effects), along with single herbs such as Scutellaria baicalensis and Pueraria lobata that target inflammatory pathways. These approaches integrate syndrome differentiation with modern pharmacological evidence to mitigate excessive inflammation and promote lung tissue repair (21, 22). *Pueraria lobata* (Kudzu) root, a traditional herbal medicine from the root of *Pueraria lobata* (Willd.) Ohwi, is effective in relieving lung dryness and cough. It is commonly used in clinical TCM to treat upper respiratory tract infections caused by heat and pathogens, pneumonia, and other illnesses (23–25), including reducing lung inflammation, combating pulmonary fibrosis, and providing antioxidant effects (26–28). Studies have shown that *Pueraria lobata* (Kudzu) root contains various bioactive compounds and is a notable source of unique and beneficial isoflavones (29, 30). Among them, Puerarin has been reported to have pharmacological protective effects against ALI (31, 32). 3’-Hydroxypuerarin (3HP), a key isoflavonoid active ingredient in *Pueraria lobata* with a high content, is a derivative of Puerarin, has higher lipid solubility (33), and exhibits significant activities in scavenging ONOO (–), NO·, and total ROS than Puerarin (34). In our preliminary experiments, we found that 3HP exhibits significant anti-inflammatory effects *in vitro*. However, studies on the pharmacoprotective effects of 3HP on acute pulmonary inflammatory responses or ALI remain limited. Therefore, based on the commonly used LPS-induced ALI model and dosage, as well as our previous research (14, 35), the current study employed an LPS (5 mg/kg) intraperitoneally injected mouse model of ALI and an *in vitro* LPS (0.5 μg/mL)-induced inflammatory damage model in RAW264.7 macrophages to evaluate the molecular pharmacologic mechanisms of 3HP. The results showed that 3HP effectively inhibited TLR4 activation, thereby suppressing NF-κB p65 signaling-mediated inflammatory cascade response and NLRP3/GSDMD signaling-mediated pyroptosis, thereby mitigating ALI. These findings provide scientific evidence for the clinical treatment of ALI and present new insights into the pharmacological role of 3HP in mitigating acute lung inflammatory diseases.

## 2 Materials and methods

### 2.1 Chemicals and reagents

3’-Hydroxypuerarin (CAS No.: 117076-54-5, Purity > 98.0%) was supplied by Weikeqi Biological Technology Co., Ltd (Sichuan, China). Resatorvid (TAK-242) (Cat No. HY-11109) and MCC950 (Cat No. HY-12815) was purchased from MedChemExpress (Shanghai, China). LPS (Cat No. L2880) was obtained from Sigma-Aldrich (Shanghai, China). Anti-IL6 (Cat No. DF6087), Anti-IκBα (Cat No. AF5002), anti-Phospho-IκBα (S32/S36) (Cat No. AF2002), anti-Caspase-8 (Cat No. AF6442), anti-ICAM1 (Cat No. AF6088), anti-VCAM1 (Cat No. DF6082), anti-COX2 (Cat No. AF7003), anti-iNOS (Cat No. AF0199), anti-Cleaved-Caspase 1 (Ala317), p10 (Cat No. AF4022), anti-GSDMD N-terminal (Cat No. DF13758), and anti-Phospho-NF-κB p65 (S536) (Cat No. AF2006) were purchased from Affinity (Affinity Biosciences, Changzhou, Jiangsu, China). Anti-TNF-α (Cat No. PY19810) was supplied by Abmart (Shanghai, China). Anti-TLR4 (Cat No. 19811-1-AP) and anti-MyD88 (Cat No. 67969-1-Ig) were obtained from Proteintech (Proteintech Group, Wuhan, Hubei, China). Anti-IL1β (Cat No. YT5201), anti-IL18 (Cat No. YN1926), anti-NF-κB p65 (Cat No. YT3108), anti-NEK7 (Cat No. YT3034), anti-NLRP3 (Cat No. YT5382), anti-ASC (Cat No. YT0365), anti-Caspase-1 (Cat No. YT5743), anti-GSDMD N-terminal (Cat No. YT7991), and anti-β-actin (Cat No. YM8343 and YM3028) were purchased from ImmunoWay (ImmunoWay Biotechnology Company, Plano, TX, USA).

### 2.2 Cell culture and cell viability

RAW264.7 macrophages were cultured at 37 °C in a 5% CO_2_ atmosphere using Dulbecco’s modified Eagle’s medium containing 10% fetal bovine serum (FBS). To assess cell viability, RAW264.7 cells (2 × 10^4/well) were seeded into 96-well plates for 24 hours, after which the cells were treated with varying concentrations of 3HP for another 24 hours. Cell viability was determined using the CCK-8 kit (GK10001) according to the manufacturer’s instructions (GLPBIO, Montclair, CA, USA).

### 2.3 Measurement of NO

RAW264.7 macrophages were plated in a 6-well plate at a density of 1 × 10^6 cells/well at 37 °C for 24 hours, followed by incubation with 3HP (25, 50, and 100 μM) and LPS (0.5 μg/ml) for an additional 24 hours. The supernatant was analyzed for NO (Cat No. S0021S) following the manufacturer’s instructions (Beyotime Biotechnology, Shanghai, China).

### 2.4 Animal experiment

*In vivo* experiments adhere to the 3R principles of animal welfare (Replacement, Reduction, Refinement). Twenty-four specific pathogen-free male C57BL/6 mice (6 to 8 weeks old, weighing 18 to 22 g) were supplied by the Guangdong Medical Laboratory Animal Center. They were housed in cages and provided free access to food and water, then used after a one-week acclimatization period. The Ethics Committee of Guangzhou University of Chinese Medicine approved all animal care and experimental procedures (No. 20240322007). Mice were randomly placed into four groups (6 mice per group): Control group (Con), LPS group (LPS), LPS + 3HP 50 mg/kg group, and LPS + 3HP 100 mg/kg group. After 1 week of oral administration of the 3HP intervention, the mice were injected intraperitoneally with LPS (5 mg/kg) to induce ALI, except in the Con group. After 24 hours of observation, serum, bronchoalveolar lavage fluid (BALF), and lung tissues were collected for further analysis.

### 2.5 Measurement of BALF protein concentration

The concentration of BALF protein was determined using the enhanced bicinchoninic acid (BCA) protein assay kit (Cat No. P0010) following the manufacturer’s instructions (Beyotime Biotechnology, Shanghai, China).

### 2.6 Enzyme-linked immunosorbent assay analysis

The levels of IL-6 (Cat No. 88-7064-86) and TNF-α (Cat No. 88-7324-86) in BALF, serum of mice and RAW264.7 macrophage supernatants were determined using ELISA kits following the manufacturer’s instructions (Invitrogen, Carlsbad, CA, USA).

### 2.7 Giemsa staining

The BALF was centrifuged to collect the supernatant, and the precipitated cells were resuspended in PBS and stained with Wright-Giemsa (Cat No. C0131). Images were acquired using a digital microscope scanner.

### 2.8 Hematoxylin and eosin staining

Lung tissues were fixed in 4% paraformaldehyde for 48 hours, dehydrated through graded ethanol solutions, embedded in paraffin blocks, and then sectioned into 5-μm-thick slices. Sections were deparaffinized, rehydrated, stained with H&E reagent (Biosharp, Hefei, China), and finally dehydrated, cleared, and mounted in neutral resin for microscopic examination. Images were acquired using a digital camera scanner.

### 2.9 Measurement of the lung wet-to-dry ratio

The W/D ratio was used to assess the severity of pulmonary edema. The left lung was collected and weighed to determine its wet (W) weight. The dry (D) weight was recorded after drying at 60 °C for 48 hours. Subsequently, the W/D ratio was then calculated *via* the following formula.


W/D ratio=Wet weight (g)/Dry weight (g)​

### 2.10 Measurement of superoxide dismutase and malondialdehyde

SOD (Cat No. A001-3-2) and MDA (Cat No. A003-1-2) levels in lung tissues were determined using commercial assay kits according to the manufacturer’s instructions (Nanjing Jiancheng Bioengineering Institute, Nanjing, China).

### 2.11 qPCR analysis

Total RNA was extracted from lung tissues with Trizol reagent (Invitrogen™, Shanghai, China). A cDNA transcription kit (Takara Biomedical Technology Co., Ltd., Dalian, China) was used for reverse transcription. Subsequently, qPCR was performed using the Bio-Rad CFX Connect Real-Time System and the TB Green™ Premix ExTaq™ kit (Takara Biomedical Technology Co., Ltd., Dalian, China). The relative mRNA levels of target genes were computed using the 2-ΔΔCt method. Primers for the genes are listed in **Supplementary Table 1**.

### 2.12 Western blotting

Lung tissues or RAW264.7 macrophages were lysed using RIPA buffer containing protease and phosphatase inhibitors for protein extraction. The protein concentration was determined using the BCA method. For immunoblotting, equal amounts of protein were separated using SDS-PAGE and subsequently transferred to a PVDF membrane. The membranes were incubated overnight at 4 °C with the specific primary antibody and for 1 hour at room temperature with the secondary antibody. Signals were detected using either the Molecular Imager^®^ System (Bio-Rad, Hercules, CA, USA) or the Amersham ImageQuant™ 800 System (Cytiva, Uppsala, Sweden) with ECL reagents. Quantification was performed using ImageJ (NIH, Bethesda, MD, USA).

### 2.13 Immunofluorescence

Lung tissue sections were deparaffinized and gradient-hydrated, incubated with 3% H_2_O_2_ prior to antigen retrieval, and then blocked with 1% BSA. For RAW264.7 macrophages, the cells were fixed with 4% paraformaldehyde, permeabilized with 0.2% Triton X-100 at room temperature, and then blocked with 1% BSA. All experimental samples were incubated overnight at 4 °C with primary antibodies, followed by an hour at room temperature with secondary antibodies, and then re-stained cell nuclei with DAPI. Images were obtained using a digital camera scanner.

### 2.14 Molecular docking analysis

The structures of the TLR4 (PDB ID: 3VQ2) target protein was acquired from the RCSB database (https://www.rcsb.org/) and processed using Schrödinger’s Protein Preparation Wizard for energy minimization and geometry optimization. The structure of 3HP was obtained from the PubChem database (https://pubchem.ncbi.nlm.nih.gov/), imported into Chem3D, optimized, and energy-minimized using the MM2 module, and saved as an SDF file for use as a ligand in molecular docking. Molecular docking was performed using the Glide module in Schrödinger Maestro (Version: 2019.01), with TLR4 binding sites predicted using the Binding Site Detection module. Docking scores of 3HP were evaluated to hypothesize its active effects.

### 2.15 Molecular dynamics simulation

To further explore how 3HP binds to the TLR4 protein at the molecular level, we used the Gromacs software (Version: 2020.6) package to perform energy minimization, heating, equilibration, and production molecular dynamics simulations of the receptor protein-small molecule complex. Protein was modeled using the AMBER99SB-ILDN force field parameters. Small-molecule ligands were constructed using the sobtop program for topology generation, with charge fitting performed *via* RESP. For the molecular dynamics simulation workflow: first, the heavy atoms of the protein and small molecule were constrained to perform 10,000 steps of energy minimization on the water molecules; then, the constraints were released, and another 10,000 steps of energy minimization were carried out on the entire system. Finally, the system underwent 100 ns of molecular dynamics simulations under the NPT ensemble. Trajectory data were saved every 20 ps and processed with the trjconv module. The binding free energies of the ligands and proteins were calculated using the gmxMMPBSA method in Gromacs 2020.6.

### 2.16 Statistical analysis

All data were expressed as mean ± SEM and analyzed with GraphPad Prism 9.0 software (San Diego, CA, USA). For data sets that satisfied the assumptions of normality and homogeneity of variances, comparisons between groups were made using one-way ANOVA. For data that were normally distributed but had unequal variances, Dunnett’s T2 test was applied. If the data did not meet the normality assumption, non-parametric tests were employed. *P*-values less than 0.05 were regarded as statistically different.

## 3 Results

### 3.1 3HP decreased the infiltration and exudation of pro-inflammatory cells in ALI mice

**Figure 1:**
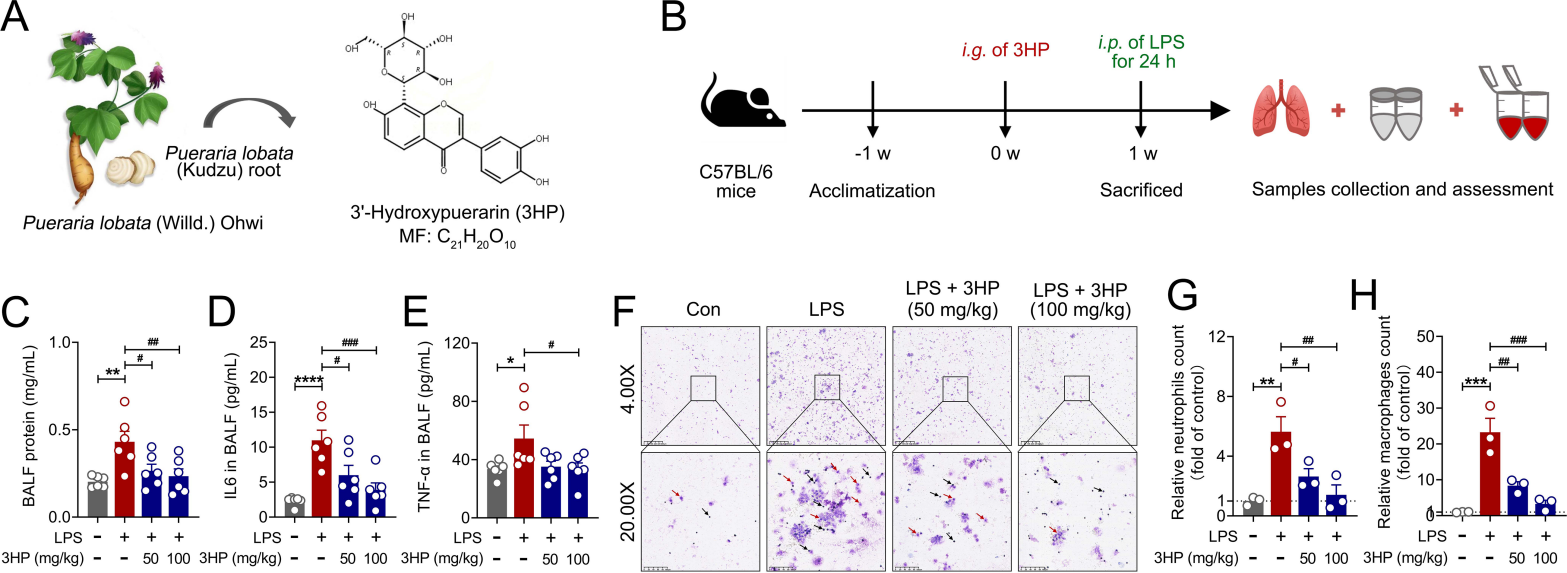
3HP reduced infiltration and exudation of pro-inflammatory cells in ALI mice. **(A)** 3HP from *Pueraria lobata* (Kudzu) root. **(B)***In vivo* experimental procedure. **(C)** BALF protein level (n = 6). **(D, E)** IL6 and TNF-α levels in BALF (n = 6). **(F)** Representative Giemsa staining in BALF, neutrophils (light purple-red, arrows: black) and macrophages (blue-purple, arrows: red), original magnification, 4.00× and 20.00×. **(G, H)** Relative neutrophils and macrophages count in BALF (n = 3). Data were expressed as mean ± SEM. Compared to the Con group, *p < 0.05, **p < 0.01, and ****p < 0.001. Compared to the LPS group, ^#^*p* < 0.05, ^##^*p* < 0.01, and ^###^*p* < 0.001.

3HP, a key isoflavonoid active ingredient in *Pueraria lobata* with a high content (**Figure 1A**), has higher lipid solubility (33). To investigate the pharmacological effect of 3HP on LPS-induced ALI in mice (**Figure 1B**), we determined the protein concentrations in BALF. As shown in **Figure 1C**, LPS stimulation increased protein leakage in the lung tissues, while 3HP intervention significantly reversed this phenomenon. We also determined the levels of inflammatory cytokines in BALF using an ELISA kit and found that 3HP significantly reduced IL6 and TNF-α levels (**Figures 1D, E**), demonstrating excellent anti-inflammatory capacity. Additionally, Giemsa staining results confirmed the effectiveness of 3HP in decreasing neutrophils (light purple-red, arrows: black) and macrophages (blue-purple, arrows: red) in BALF (**Figures 1F–H**), indicating that 3HP positively affects the reduction of pro-inflammatory cell infiltration and exudation in ALI mice.

### 3.2 3HP alleviated LPS-induced ALI in mice by suppressing inflammatory cascade responses

**Figure 2:**
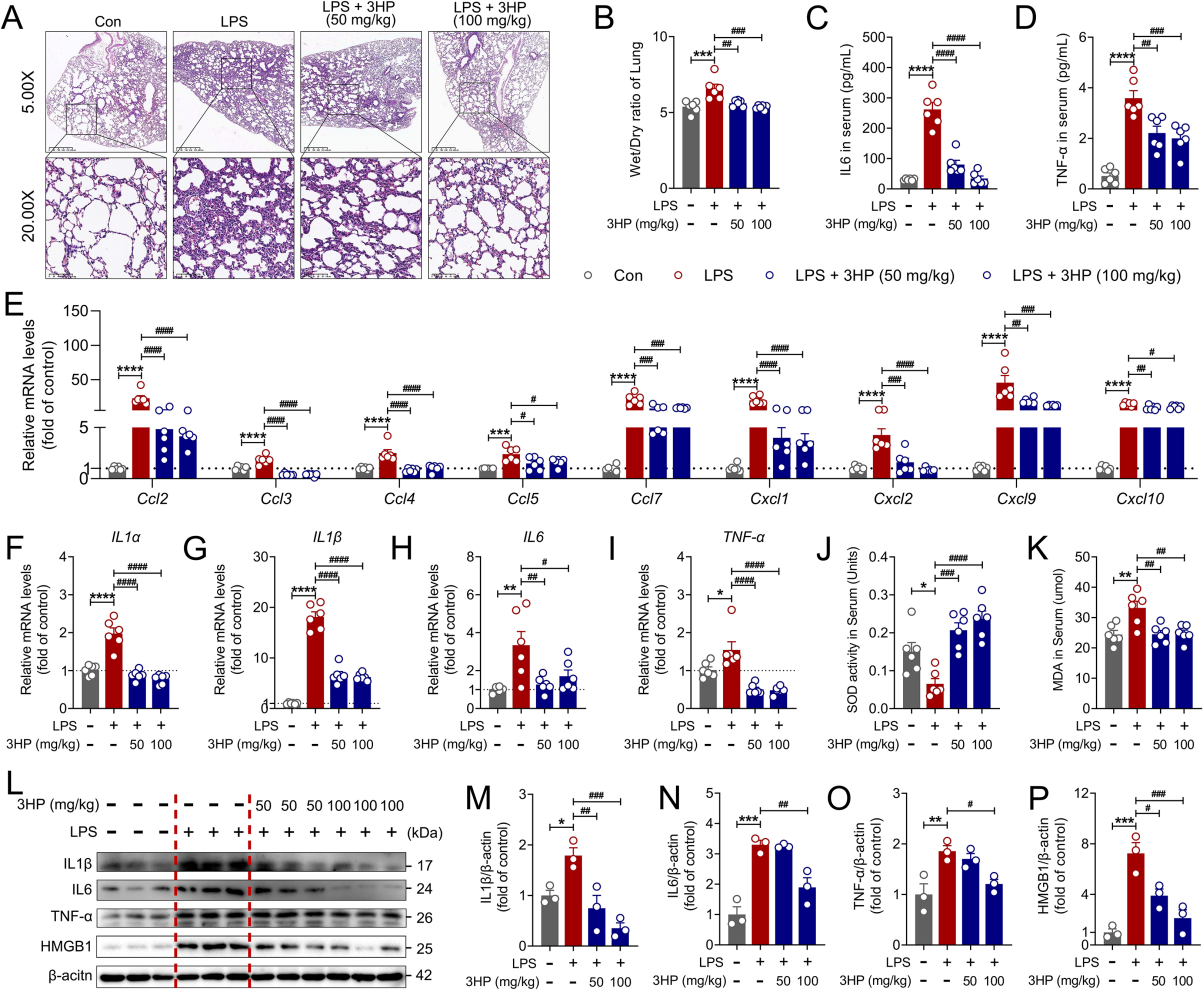
3HP alleviated LPS-induced ALI in mice by suppressing inflammatory cascade responses. **(A)** Representative H&E staining in lung tissues, original magnification, 5.00× and 20.00×. **(B)** W/D radio of the lungs (n = 6). **(C, D)** IL6 and TNF-α levels in serum (n = 6). **(E)** mRNA levels of *Ccl2*, *Ccl3*, *Ccl4*, *Ccl5*, *Ccl7*, *Cxcl1*, *Cxcl2*, *Cxcl9*, and *Cxcl10* in lung tissues (n = 6). **(F–I)** mRNA levels of *IL1α*, *IL1β*, *IL6*, and *TNF-α* in lung tissues (n = 6). **(J, K)** SOD and MDA levels in serum (n = 6). **(L–P)** Representative immunoblotting and quantification of IL1β, IL6, TNF-α, and HMGB1 in lung tissues (n = 3). Data were expressed as mean ± SEM. Compared to the Con group, *p < 0.05, **p < 0.01, ***p < 0.001, and ****p < 0.00. Compared to the LPS group, ^#^*p* < 0.05, ^##^*p* < 0.01, ^###^*p* < 0.001, and ^####^*p* < 0.0001.

The immune cell-mediated inflammatory response plays a crucial role in regulating the progression of ALI (36). LPS-induced lung tissues in ALI mice displayed significant infiltration of inflammatory cells and pulmonary edema, while prophylactic administration of 3HP notably reduced lung inflammatory injury (**Figure 2A**). The lung W/D weight ratio clearly confirmed this result (**Figure 2B**). Additionally, we analyzed serum inflammatory cytokine levels using the ELISA assay and found that 3HP significantly reduced IL-6 and TNF-α levels (**Figures 2C, D**). Further qPCR analysis indicated that LPS stimulation increased the levels of chemokines and inflammatory cytokines, while 3HP significantly reduced mRNA levels of *Ccl2*, *Ccl3*, *Ccl4*, *Ccl5*, *Ccl7*, *Cxcl1*, *Cxcl2*, *Cxcl9*, *Cxcl10*, *IL1α*, *IL1β*, *IL6*, and *TNF-α* (**Figures 2E–I**). Interestingly, we also found that 3HP significantly increased SOD activity and reduced MDA levels (**Figures 2J, K**), indicating its ability to resist oxidative stress. Moreover, the impressive anti-inflammatory potential of 3HP was reaffirmed by immunoblotting results, which demonstrated that 3HP markedly inhibited the expression of IL1β, IL6, TNF-α, and HMGB1 proteins in the lung tissues of ALI mice (**Figures 2L–P**). All these findings suggested that 3HP attenuated ALI by inhibiting LPS-stimulated acute inflammatory responses in the lungs.

### 3.3 3HP alleviated LPS-induced ALI in mice by inhibiting TLR4/NF-κB p65 signaling

**Figure 3:**
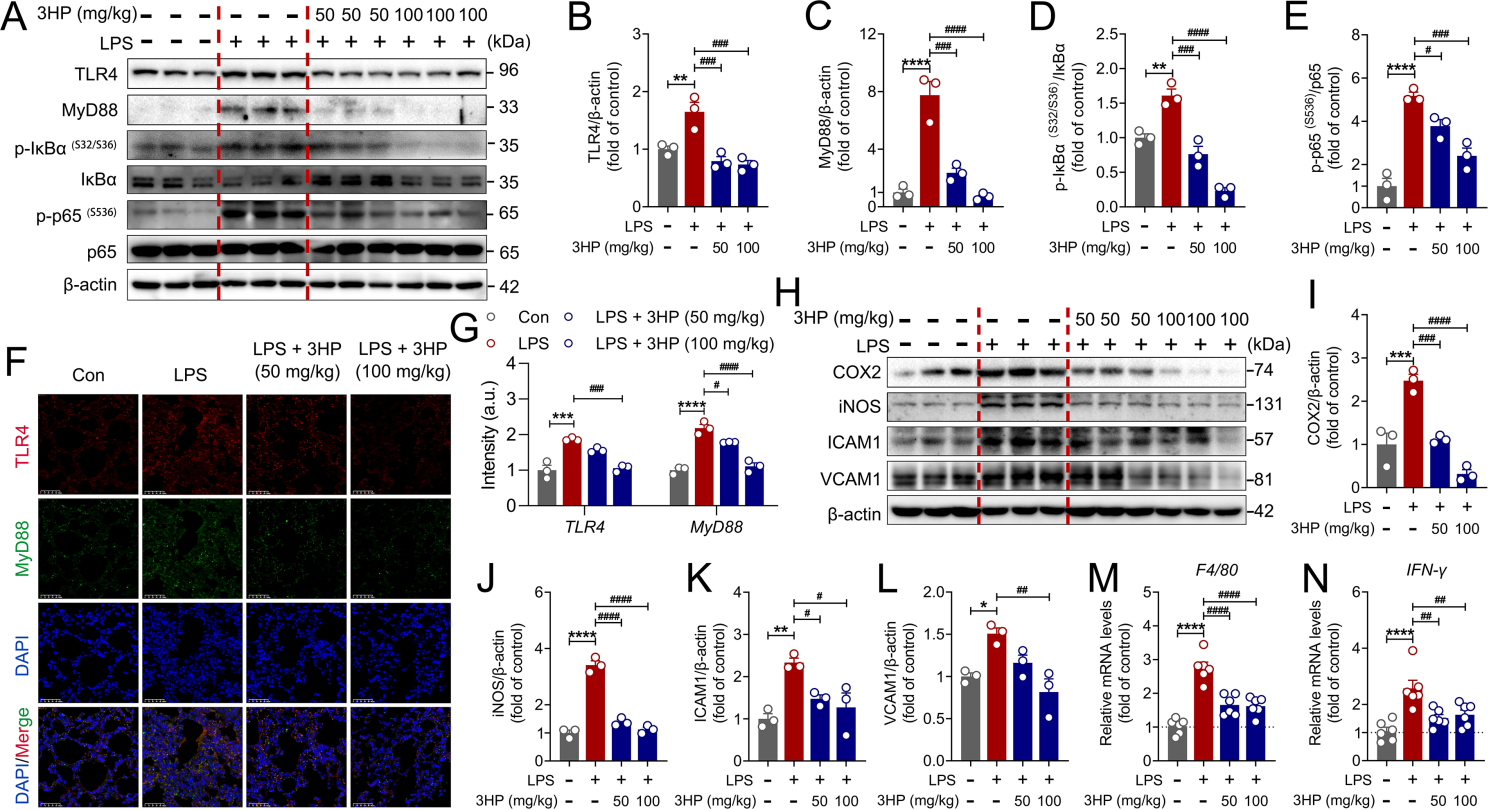
3HP alleviated LPS-induced ALI in mice by inhibiting TLR4/NF-κB p65 signaling. **(A–E)** Representative immunoblotting and quantification of TLR4, MyD88, p-IκBα (S32/S36), IκBα, p-NF-κB p65 (S536), and NF-κB p65 in lung tissues (n = 3). **(F, G)** Representative immunofluorescence images and intensity of TLR4-positive and MyD88-positive in lung tissues (n = 3). Scale bars: 25 μm. **(H–L)** Representative immunoblotting and quantification of COX2, iNOS, ICAM1, and VCAM1 in lung tissues (n = 3). **(M, N)** mRNA levels of *F4/80* and *IFN-γ* in lung tissues (n = 6). Data were expressed as mean ± SEM. Compared to the Con group, *p < 0.05, **p < 0.01, ***p < 0.001, and ****p < 0.000. Compared to the LPS group, ^#^*p* < 0.05, ^##^*p* < 0.01, ^###^*p* < 0.001, and ^####^*p* < 0.0001.

It has long been established that NF-κB signaling plays a crucial role in the pathogenesis of ALI (37). To investigate the effect of 3HP on LPS-activated NF-κB signaling, we examined the expression levels of the core components using immunoblotting. As shown in **Figures 3A–E**, 3HP significantly reduced the expression levels of LPS-stimulated TLR4, MyD88, p-IκBα (S32/S36), and p-NF-κB p65 (S536). Additionally, immunofluorescence analysis further confirmed the inhibitory effect of 3HP on LPS-activated TLR4 and MyD88 (**Figures 3F, G**). Notably, NF-κB signaling activation and transactivation significantly regulated the transcription of inflammatory mediators, thereby exacerbating the inflammatory cascade response. Thus, we further analyzed biomarkers of the inflammatory response and found that 3HP markedly inhibited the expression of COX2, iNOS, ICAM1, and VCAM1 (**Figures 3H–L**). Additionally, qPCR analysis indicated that 3HP significantly down-regulated the mRNA levels of *F4/80* and *IFN-γ* (**Figures 3M, N**), reaffirming its strong anti-inflammatory pharmacological effects. Overall, these results demonstrated that 3HP alleviated LPS-induced ALI in mice by inhibiting TLR4/NF-κB p65 signaling pathway.

### 3.4 3HP alleviated LPS-stimulated ALI in mice by inhibiting NLRP3 inflammasome activation

**Figure 4:**
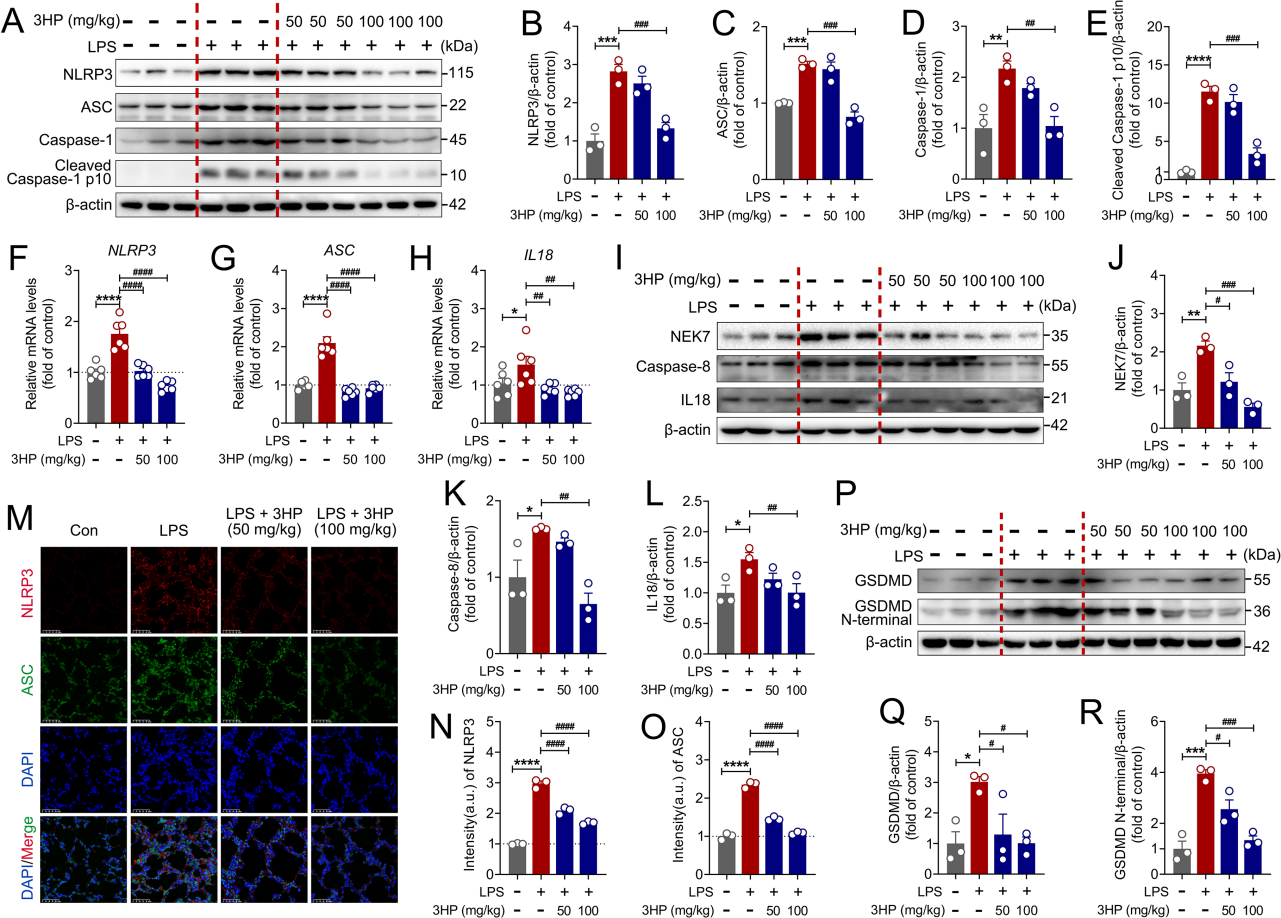
3HP alleviates LPS-stimulated ALI in mice by inhibiting NLRP3 inflammasome activation. **(A–E)** Representative immunoblotting and quantification of NLRP3, ASC, Caspase-1, and Cleaved Caspase-1 p10 in lung tissues (n = 3). **(F–H)** mRNA levels of *NLRP3*, *ASC*, and *IL18* in lung tissues (n = 6). **(I–L)** Representative immunoblotting and quantification of NEK7, Caspase-8, and IL18 in lung tissues (n = 3). **(M–O)** Representative immunofluorescence images and intensity of NLRP3-positive and ASC-positive in lung tissues (n = 3). Scale bars: 25 μm. **(P–R)** Representative immunoblotting and quantification of GSDMD and GSDMD N-terminal in lung tissues (n = 3). Data were expressed as mean ± SEM. Compared to the Con group, *p < 0.05, **p < 0.01, ***p < 0.001, and ****p < 0.0001. Compared to the LPS group, ^#^*p* < 0.05, ^##^*p* < 0.01, ^###^*p* < 0.001, and ^####^*p* < 0.000.

To further explore the molecular mechanisms of 3HP in alleviating ALI, we concentrated on assessing the pharmacological effects of 3HP on NLRP3 signaling. As illustrated in **Figures 4A–E**, LPS stimulation activated the NLRP3 inflammasome, leading to the upregulation of NLRP3, ASC, Caspase-1, and Cleaved Caspase-1 p10. Interestingly, 3HP significantly reversed this phenomenon. Additionally, qPCR results confirmed the inhibitory effect of 3HP on the mRNA levels of the *NLRP3*, *ASC*, and *IL18* (**Figures 4F–H**). Notably, we also found that 3HP significantly reduced the expression of NEK7, Caspase-8, and IL18 (**Figures 4I–L**). Immunofluorescence confirmed the pharmacological inhibition of NLRP3 activation by 3HP (**Figures 4M–O**). Indeed, the NLRP3 inflammasome activates Caspase-1, which cleaves GSDMD. In this study, we found that 3HP significantly inhibited the expression of GSDMD and GSDMD N-terminal (**Figures 4P–R**), highlighting the substantial inhibitory effect of 3HP on NLRP3 signaling. Taken together, these findings suggested that 3HP mitigated LPS-induced ALI in mice by inhibiting the NLRP3/GSDMD-mediated cellular pyroptosis.

### 3.5 3HP attenuated LPS-stimulated acute inflammatory injury in RAW264.7 macrophages by inhibiting inflammatory cascade responses

**Figure 5:**
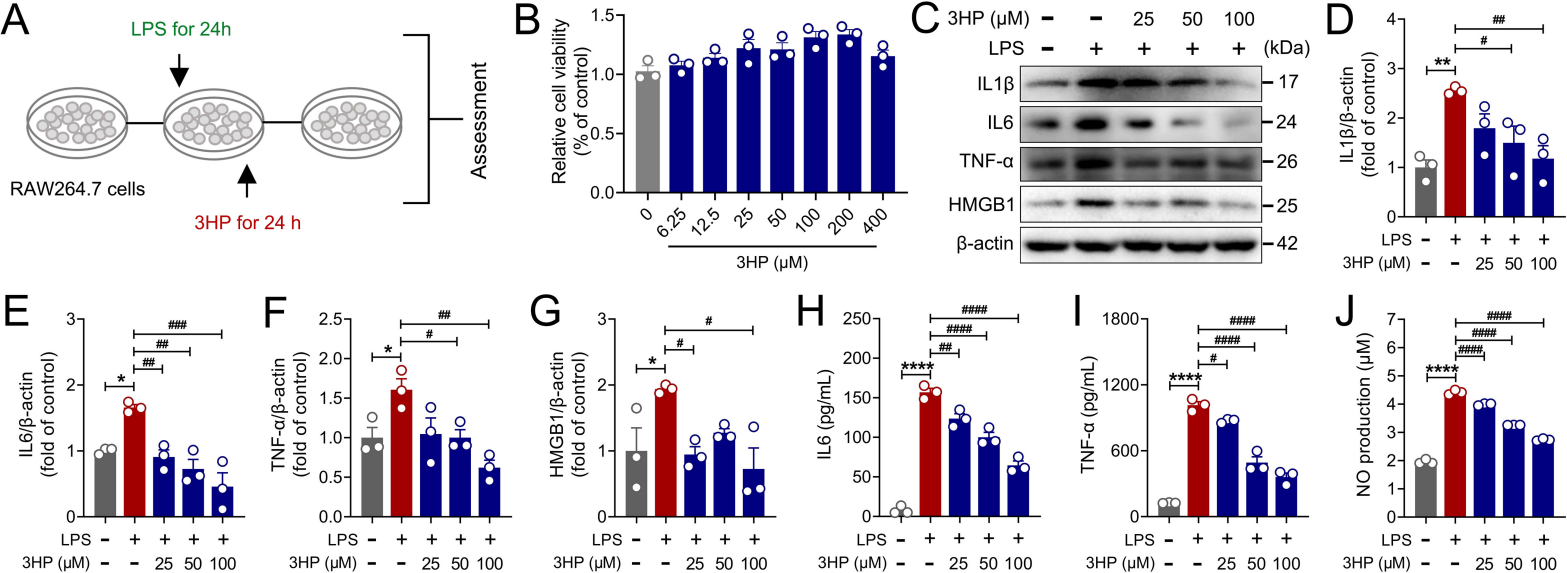
3HP attenuated LPS-stimulated acute inflammatory injury in RAW264.7 macrophages by inhibiting inflammatory cascade responses. **(A)***In vitro* experimental procedure. **(B)** Relative cell viability. **(C–G)** Representative immunoblotting and quantification of IL1β, IL6, TNF-α, and HMGB1 in RAW264.7 macrophages. **(H–J)** IL6, TNF-α, and NO levels in supernatants of RAW264.7 macrophages. Data were expressed as mean ± SEM (n = 3). Compared to the Con group, *p < 0.05, **p < 0.01, and ****p < 0.0001. Compared to the LPS group, ^#^*p* < 0.05, ^##^*p* < 0.01, ^###^*p* < 0.001, and ^####^*p* < 0.0001.

Research indicates that pathogens enter lung tissues and activate alveolar macrophages, leading to the release of significant amounts of inflammatory factors, which consequently trigger an inflammatory cascade response (38). In this study, we utilized an *in vitro* LPS-stimulated RAW264.7 macrophage model of acute inflammatory injury to evaluate the pharmacological effects of 3HP (**Figure 5A**). As shown in **Figure 5B**, the CCK-8 assay demonstrated that various concentrations of 3HP (6.25, 12.5, 25, 50, 100, 200, and 400 μM) exhibited no toxic effects on RAW264.7 macrophages. Interestingly, it also showed a slight promotional impact on cell proliferation. Further immunoblotting assays showed that 3HP (25, 50, and 100 μM) significantly reduced the expression of inflammatory cytokines IL1β, IL6, TNF-α, and HMGB1 stimulated by LPS (**Figures 5C–G**). Additionally, we found that 3HP significantly reduced IL6, TNF-α, and NO levels in LPS-stimulated RAW264.7 macrophage supernatants (**Figures 5H–J**), reaffirming its strong anti-inflammatory response capabilities.

### 3.6 3HP attenuated LPS-stimulated inflammatory injury in RAW264.7 macrophages by inhibiting TLR4/NF-κB p65 signaling

**Figure 6:**
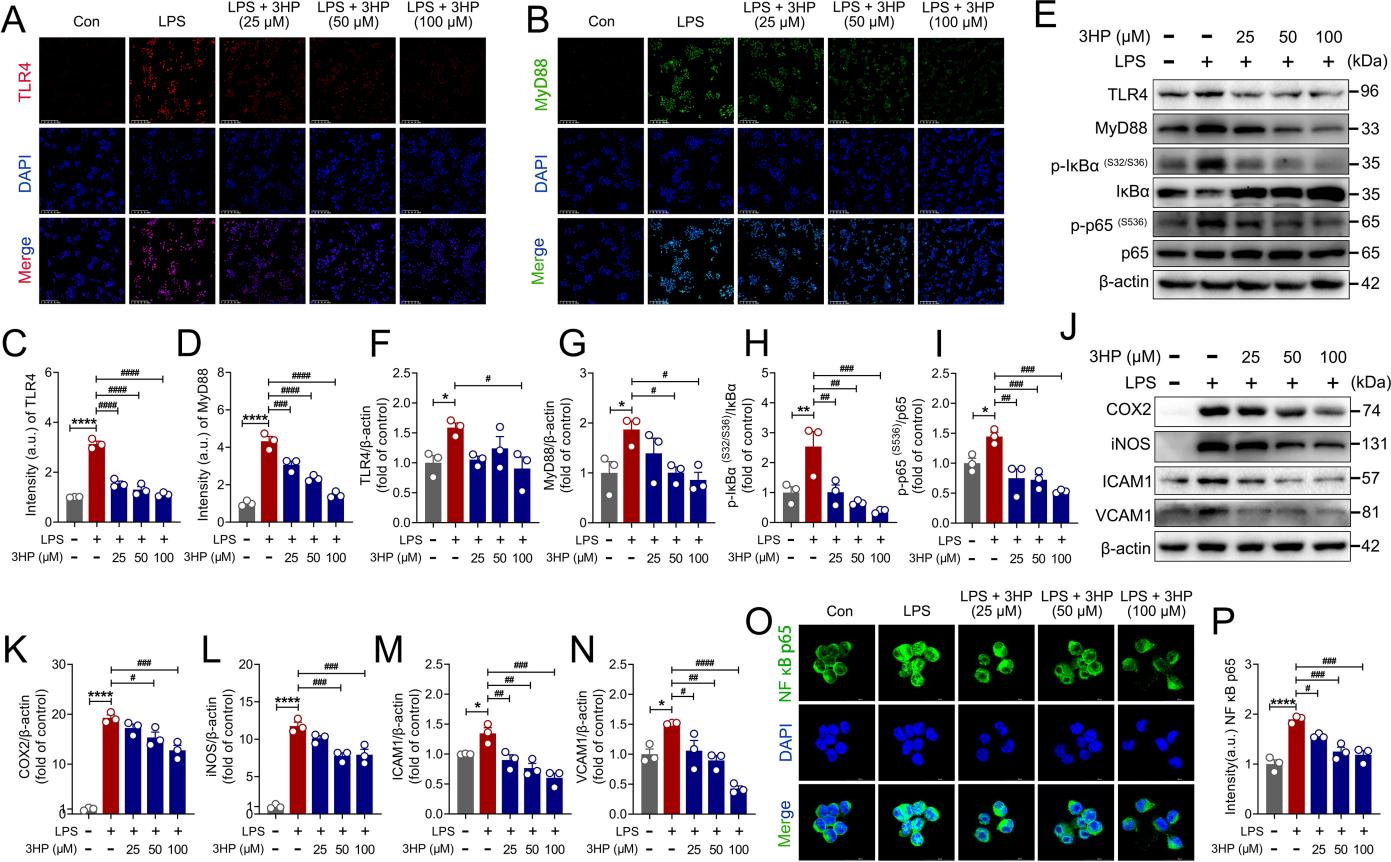
3HP attenuated LPS-stimulated inflammatory injury in RAW264.7 macrophages by inhibiting TLR4/NF-κB p65 signaling. **(A–D)** Representative immunofluorescence images and intensity of TLR4-positive and MyD88-positive in RAW264.7 macrophages, Scale bars: 100 μm. **(E–I)** Representative immunoblotting and quantification of TLR4, MyD88, p-IκBα (S32/S36), IκBα, p-NF-κB p65 (S536), and NF-κB p65 in RAW264.7 macrophages. **(J–N)** Representative immunoblotting and quantification of COX2, iNOS, ICAM1, and VCAM1 in RAW264.7 macrophages. **(O–P)** Representative immunofluorescence images and intensity of NF-κB p65 translocation into the nucleus. Data were expressed as mean ± SEM (n = 3). Compared to the Con group, *p < 0.05, **p < 0.01, and ****p < 0.0001. Compared to the LPS group, ^#^*p* < 0.05, ^##^*p* < 0.01, ^###^*p* < 0.001, and ^####^*p* < 0.0001.

LPS, a key element of the outer membrane of Gram-negative bacteria, activates intracellular signaling pathways, such as MyD88 and interleukin-1 receptor-associated kinase (IRAK), by binding to the TLR4 receptor of the host cell. This ultimately activates the NF-κB transcription factor, which initiates the production and release of inflammatory factors (11). Based on the findings above, we reanalyzed the effect of 3HP on the TLR4/NF-κB pathway in an *in vitro* model of acute inflammatory injury. Our analysis revealed that 3HP significantly inhibited the fluorescence expression of TLR4 and MyD88 in LPS-stimulated RAW264.7 macrophages (**Figures 6A–D**). Further immunoblot analysis demonstrated that 3HP significantly reduced the expression of key components of NF-κB signaling, including TLR4, MyD88, p-IκBα (S32/S36), and p-NF-κB p65 (S536) (**Figures 6E–I**). Furthermore, we also discovered that 3HP significantly inhibited the expression of the inflammatory mediators COX2, iNOS, ICAM1, and VCAM1 (**Figures 6J–N**). In fact, the translocation of p65/p50 into the nucleus is an important factor in the activation of the classic NF-κB signaling. Therefore, we analyzed this phenomenon using immunofluorescence analysis. The results revealed that 3HP suppressed NF-κB p65 translocation from the cytoplasm to the nucleus (**Figures 6O, P**). Overall, these results indicated that 3HP inhibited LPS-stimulated TLR4 activation, thereby reducing IκBα phosphorylation and degradation, and blocking the translocation of the active NF-κB p65 transcription factor into the nucleus, thereby inhibiting target gene expression. Interestingly, these findings aligned with the results of the *in vivo* experiments mentioned above, further emphasizing 3HP’s pharmacological inhibition of NF-κB signaling in ameliorating ALI.

### 3.7 3HP alleviated LPS-stimulated acute inflammatory injury in RAW264.7 macrophages by inhibiting NLRP3/GSDMD signaling-mediated cellular pyroptosis

**Figure 7:**
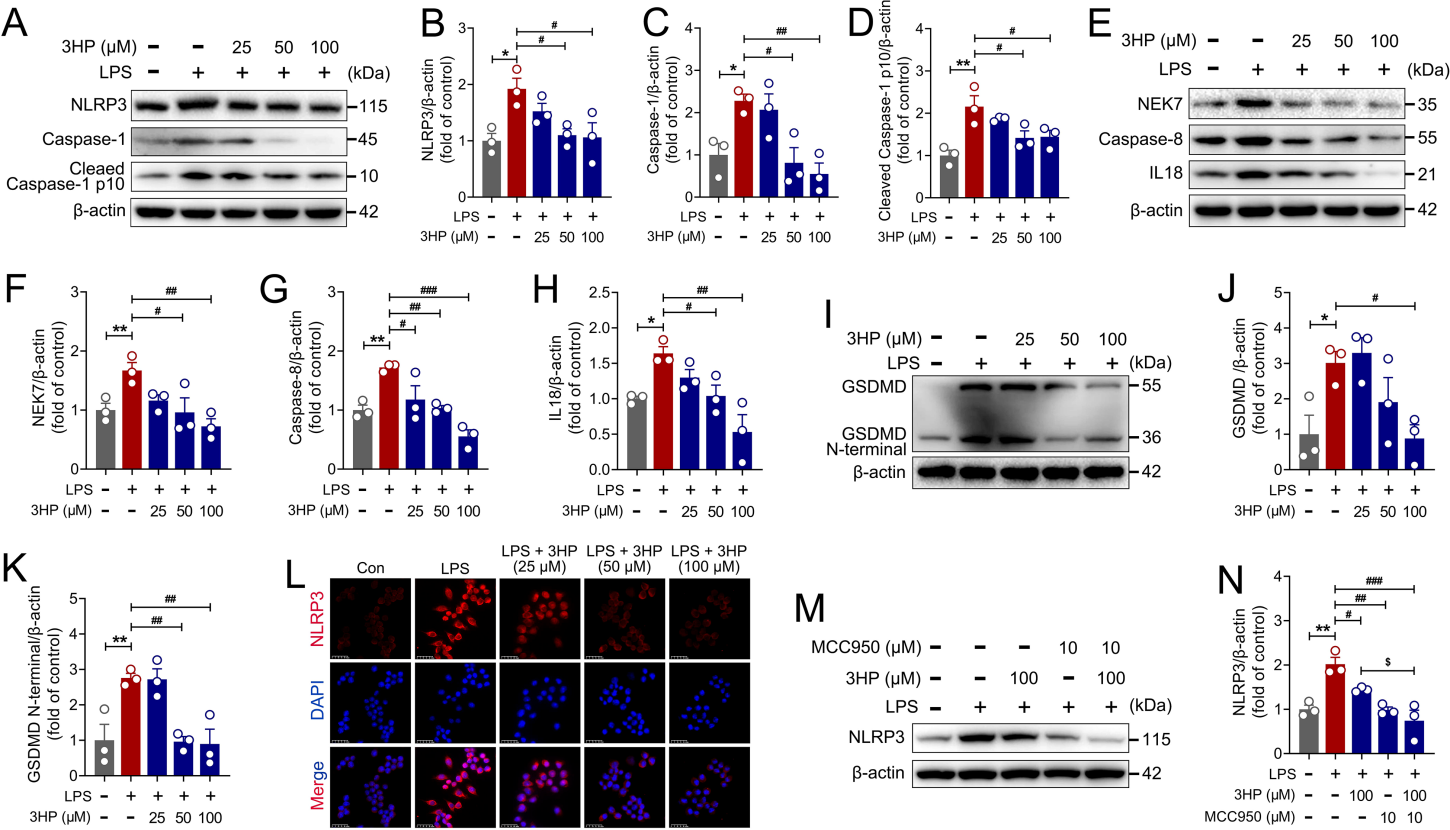
3HP alleviated LPS-stimulated acute inflammatory injury in RAW264.7 macrophages by inhibiting NLRP3/GSDMD signaling-mediated cellular pyroptosis. **(A–D)** Representative immunoblotting and quantification of NLRP3, Caspase-1, and Cleaved Caspase-1 p10 in RAW264.7 macrophages. **(E–H)** Representative immunoblotting and quantification of NEK7, Caspase-8, and IL18 in RAW264.7 macrophages. **(I–K)** Representative immunoblotting and quantification of GSDMD and GSDMD N-terminal in RAW264.7 macrophages. **(L)** Representative immunofluorescence images of NLRP3-positive in RAW264.7 macrophages, Scale bars: 25 μm. **(M, N)** Representative immunoblotting and quantification of NLRP3 after MCC950 and 3HP intervention in RAW264.7 macrophages. Data were expressed as mean ± SEM (n = 3). Compared to the Con group, *p < 0.05, and **p < 0.01. Compared to the LPS group, ^#^*p* < 0.05, ^##^*p* < 0.01, and ^###^*p* < 0.001. Compared to the 3HP group, ^$^*p* < 0.05.

The above *in vivo* experiments have revealed that 3HP exerts a pharmacological inhibitory effect on the activation of NLRP3 inflammasome in lung tissues of ALI mice. To further confirm the ameliorative effect of 3HP on acute inflammatory injury, we analyzed key proteins involved in NLRP3 inflammasome activation by immunoblotting in LPS-stimulated RAW264.7 macrophages. As shown in **Figures 7A–H**, 3HP significantly decreased the expression of NLRP3, Caspase-1, Cleaved Caspase-1 p10, NEK7, Caspase-8, and IL18. Interestingly, we also found that 3HP significantly inhibited the cleavage of the substrate GSDMD by Caspase-1 and decreased the membrane-pore-forming activity of the GSDMD N-terminal domain, thereby reducing cellular pyroptosis (**Figures 7I–K**). Further immunofluorescence results indicated that 3HP significantly suppressed NLRP3 expression (**Figure 7L**), reaffirming its pharmacological inhibitory effect on NLRP3 inflammasome activation. Additionally, we selected MCC950, a specific inhibitor of NLRP3 inflammasome activation, to further evaluate the effects of 3HP and MCC950. As shown in **Figures 7M, N**, MCC950 enhanced the LPS-stimulated pharmacological inhibition of NLRP3 by 3HP, emphasizing 3HP’s protective effect against LPS-stimulated cellular acute inflammatory injury. All these findings indicated that 3HP could reduce ALI by inhibiting NLRP3/GSDMD signaling-mediated pyroptosis.

### 3.8 3HP might ameliorate ALI by inhibiting NF-κB/NLRP3 signaling through targeting TLR4

**Figure 8:**
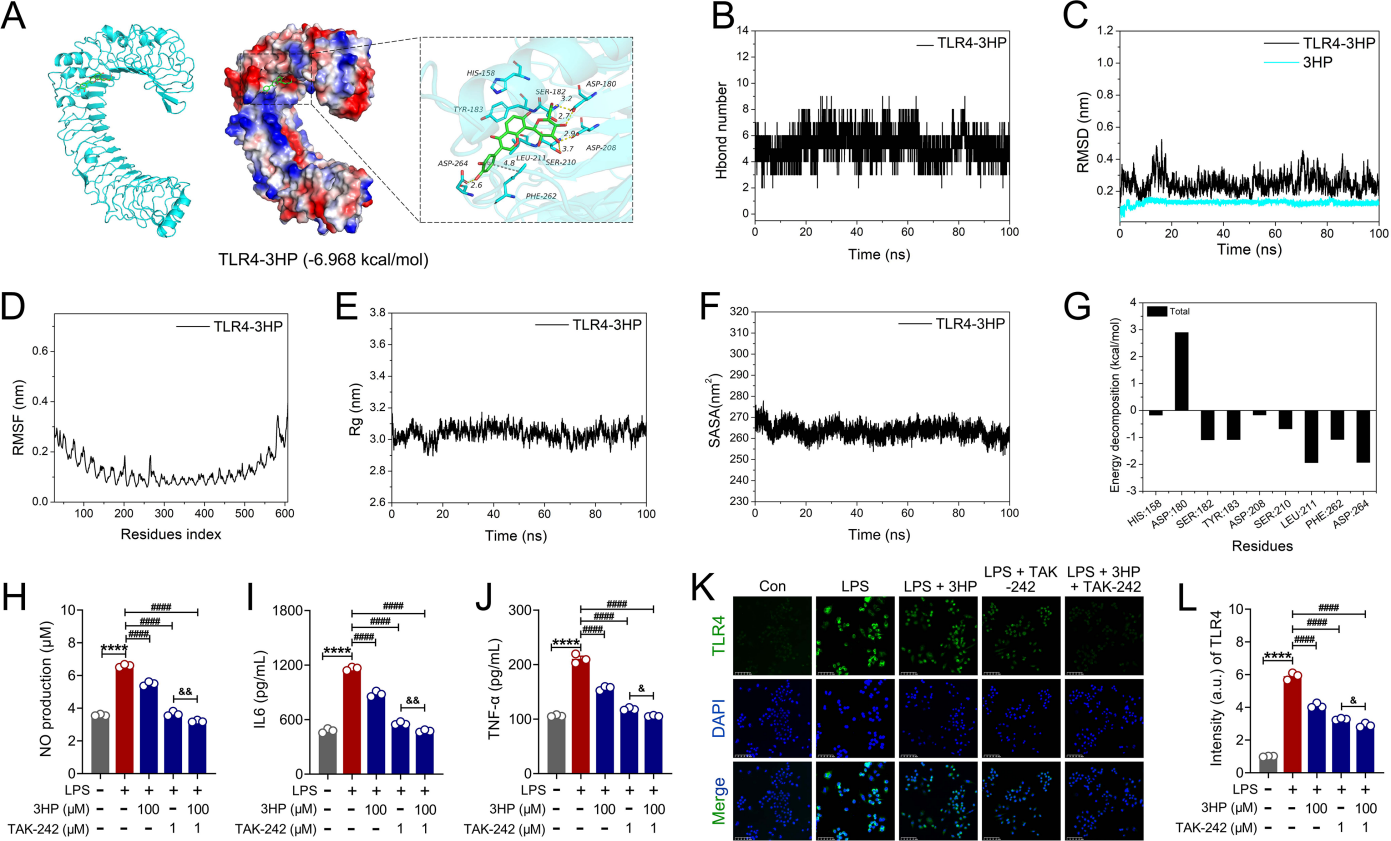
3HP bound to TLR4 stably. **(A)** The binding mode of TLR4 with 3HP. (The 3D structure of complex. The electrostatic surface of protein. The detail binding mode of complex. The backbone of protein was rendered in tube and colored in green. Compound is rendering by green). **(B)** The hydrogen bond number of 3HP with TLR4. **(C)** The RMSD of 3HP with TLR4. **(D)** The RMSF of 3HP with TLR4. **(E)** The Rg of 3HP with TLR4. **(F)** The SASA of 3HP with TLR4. **(G)** The energy decomposition of binding free energy for TLR4-3HP. **(H–J)** levels of IL6, TNF-α, and NO (n = 3). **(K, L)** Representative immunofluorescence images and intensity of TLR4-positive in RAW264.7 macrophages (n = 3). Scale bars: 50 μm.

The pharmacological effects of 3HP in inhibiting TLR4/NF-κB and NLRP3/GSDMD signaling to alleviate LPS-induced ALI have been demonstrated. Indeed, in LPS-stimulated activation of the classical NF-κB signaling pathway, upstream TLR4 activation plays a crucial role. To investigate the pharmacological molecular mechanism by which 3HP inhibits TLR4, we conducted molecular docking analysis. As shown in **Figure 8A**, the molecular binding energy of 3HP to TLR4 was -6.968 kcal/mol (Less than -5.0 kcal/mol), indicating that 3HP has a strong affinity for TLR4 and can bind stably to exert pharmacological inhibitory activity.

To gain a more intuitive understanding of TLR4 binding to 3HP, we used molecular dynamics simulations to track changes in the number of hydrogen bonds between the protein and the small molecule throughout the simulation. As shown in **Figure 8B**, 3HP was able to form about five hydrogen bonding interactions with the TLR4 pocket amino acids (100% hydrogen bond occupancy), and these hydrogen bonds played a key role in stabilizing the 3HP-TLR4 binding. Furthermore, the average root mean square deviation (RMSD) of the complexes were all below 0.3 nm, and they generally reached dynamic equilibrium around 40 ns. Interestingly, we observed that the fluctuations of 3HP were significantly smaller than the RMSD fluctuations of the TLR4 protein, suggesting that the compounds could consistently remain in the protein’s binding site (**Figure 8C**). Meanwhile, the root mean square fluctuation (RMSF) results indicated that a few amino acids in the complex formed by the interaction between TLR4 and 3HP experienced significant conformational changes, especially near the C-terminal and N-terminal sites. This was mainly because these regions are at the ends of the protein, making their conformation more prone to specific changes during the simulation (**Figure 8D**). To examine the relative densities and stability of complexes formed by primary and secondary site binding, we measured the radius of gyration (Rg) of the target proteins and observed that TLR4 Rg initially increased slightly before decreasing. However, binding of TLR4 to 3HP enhanced the formation of more effective interactions within the protein, thereby increasing the stability of the complexes (**Figure 8E**). Moreover, the solvent-accessible surface area (SASA) begins to decrease after the kinetic adjustment, which is beneficial for complex stability (**Figure 8F**). Notably, the binding free energy is a key tool for analyzing changes in the ligand binding mode by measuring the ligand’s thermodynamic properties. Negative values of the binding free energy (ΔGbinding energy) indicate system stability, while positive values suggest instability. As shown in **Supplementary Table 2**, electrostatic interactions play a significant role in stabilizing small molecule compounds, followed by van der Waals forces. The binding free energy of 3HP with TLR4 protein was -33.48 ± -5.71 kcal/mol, mainly due to hydrogen-bonding interactions between the compounds and the protein pockets, making electrostatic interactions a key contributor to the compounds’ stability (-85.01 ± -5.4 kcal/mol). Additionally, van der Waals forces also significantly contribute (-22.46 ± 0.87 kcal/mol), indicating that 3HP can remain stably in TLR4 pocket with strong van der Waals interactions with surrounding residues. We further examined the binding free energies and found that the amino acids HIS:158, ASP:180, SER:182, TYR:183, ASP:208, SER:210, LEU:211, PHE:262, and ASP:264 play a significant role in stabilizing 3HP (**Figure 8G**). In summary, 3HP shows strong affinity for TLR4. These affinities can help form small molecules into stable complexes with the protein, thereby exerting active inhibitory effects.

To further evaluate the pharmacological effects of 3HP on TLR4, we analyzed the therapeutic impact of TAK-242, a TLR4 inhibitor. Results revealed that TAK-242 potentiated the pharmacological inhibition of TLR4 by 3HP, as evidenced by significantly reduced levels of NO, IL6, and TNF-α (**Figures 8H–J**), along with decreased TLR4 expression (**Figures 8K, L**). This indicates that 3HP partially exerts its inhibitory effect on downstream inflammatory cascades by modulating TLR4.

## 4 Discussion

ALI, a prevalent respiratory critical illness characterized by high morbidity and mortality rates, is caused by various pathogenic factors both within and outside the lungs (1, 2). An imbalance in the inflammatory response cascade caused by infection and trauma is the root cause of ALI progression (39, 40). While significant breakthroughs have been achieved in ALI/ARDS research, no effective drug therapies currently exist. Therefore, exploring pharmacological treatments for ALI/ARDS is urgently needed. LPS, a significant component of the outer membrane of Gram-negative bacteria, is an endotoxin commonly used to induce various inflammatory models, such as ALI. Once inside the host, LBP recognizes LPS and binds to transport to the surface of myeloid-derived cells to bind to mCD14, forming the LPS-LBP-CD14 triplex, which is subsequently transported to the TLR4-MD2 protein complex. The triplex complex binds to TLR4 with the assistance of MD-2 and activates TLR4, thereby activating intracellular classical NF-κB signaling pathway, leading to the release of inflammatory mediators such as IL1β, IL6, TNF-α, and NO, and ultimately triggering an inflammatory cascade response. It is important to note that NF-κB signaling has long been recognized as a significant factor in the progression of ALI (37). Thus, pharmacologically inhibiting the core target proteins of NF-κB signaling is an essential way to ameliorate ALI. In fact, natural compounds have consistently inspired drug development, with many natural products demonstrating potential therapeutic effects for ALI/ARDS.

TCM, a healthcare system that has evolved over thousands of years for the Chinese people, has been extensively utilized to treat various diseases (41). An increasing number of active ingredients and formulations of TCM have been demonstrated to have significant therapeutic effects in treating ALI, which provides some guidance for clinical practice (22). *Pueraria lobata* (Kudzu) root, a traditional Chinese herb mentioned in the TCM, is known for its effectiveness in clearing heat, generating fluids, quenching thirst, and alleviating lung dryness and cough. It treats upper respiratory tract infections caused by heat and pathogens, pneumonia, and various other illnesses (23–25). Modern pharmacological studies have confirmed its ability to reduce lung inflammation, combat pulmonary fibrosis, and exert an anti-oxidative stress effect (26–28). *Pueraria lobata* (Kudzu) root is rich in isoflavonoid active compounds, such as puerarin and 3’-hydroxypuerarin (3HP). 3HP is a derivative of puerarin, has higher lipid solubility than puerarin (33), and exhibits significant ONOO (-), NO-, and total ROS scavenging activity (34). Studies have consistently shown that puerarin alleviates ALI by suppressing the inflammatory response and regulating NLRP3 inflammasome-induced cellular pyroptosis (31, 32). However, the effects and mechanisms of 3HP in alleviating acute inflammatory lung injury remain unclear.

In the current study, we evaluated the pharmacological benefits of 3HP through an *in vivo* LPS-induced ALI in mice and an *in vitro* LPS-induced acute inflammatory injury in the RAW264.7 macrophage model. Previous extensive research has demonstrated that intratracheal or intraperitoneal LPS stimulation can significantly induce acute inflammatory injury in mouse lungs (9). Concurrently, LPS-induced inflammatory injury in RAW 264.7 cells serves as a commonly used *in vitro* model for investigating ALI (10). This cell line produces a robust, prototypical inflammatory response, particularly upon stimulation with agents such as lipopolysaccharide (a TLR4 agonist). TLR4 stimulation activates intracellular signaling cascades, mobilizing nuclear transcription factors like nuclear factor-κB, ultimately leading to the production of inflammatory mediators including NO, TNF-α, ILβ, and IL6. As expected, we found that 3HP, an active isoflavone ingredient derived from the root of *Pueraria lobata* (Kudzu), significantly inhibited endotoxin LPS-stimulated TLR4 activation, decreased the expression of MyD88, a key transducer protein in intrinsic immune signaling, reduced downstream NF-κB pathway signaling, suppressed IκBα protein phosphorylation and protease degradation, and blocked the translocation of active NF-κB p65 into the nucleus. This ultimately inhibited the expression of target genes, including chemokines such as *Ccl2*, *Ccl3*, *Ccl4*, *Ccl5*, *Ccl7*, *Cxcl1*, *Cxcl2*, *Cxcl9*, *Cxcl10*, and inflammatory factors such as *IL1α*, *IL1β*, *IL6*, *TNF-α*, *F4/80*, and *IFN-γ*. These findings first highlight the remarkable ability of 3HP to reduce the inflammatory damage caused by endotoxin infection, indicating that it can attenuate LPS-induced ALI both *in vitro* and *in vivo* by inhibiting the inflammatory cascade response mediated by the classical TLR4/NF-κB p65 pathway signaling.

Indeed, the endotoxin-activated TLR4/NF-κB signaling pathway enhances the transcription and translation of NLRP3 and cytokines, triggers changes in the post-translational modifications of NLRP3, and thereby mediates the assembly of inflammasomes (42). NLRP3, a pattern recognition receptor (PRR) that triggers an inflammatory cascade response by recognizing pathogen-associated molecular patterns (PAMPs) and damage-associated molecular patterns (DAMPs), serves as a critical modulator of innate immune defense (43). Activating the NLRP3 inflammasome is crucial in the progression of ALI (18, 19). Previous studies have demonstrated that Natural product derived phytochemicals or herbal formulations can alleviate ALI by decreasing the inhibition of NLRP3 inflammasome activation (44). For instance, dihydromyricetin alleviates sepsis-induced acute lung injury through Inhibiting NLRP3 inflammasome-dependent pyroptosis in mice model (45). Sinensetin ameliorates LPS-induced acute lung injury by suppressing Txnip/NLRP3/Caspase-1/GSDMD signaling-mediated inflammatory responses and pyroptosis (14). In this study, we found that 3HP significantly inhibited NLRP3 inflammasome assembly and activation, demonstrating consistent pharmacological inhibition of NLRP3 inflammasome activation. Notably, NEK7 is a crucial mediator of NLRP3 activation (46). Intracellular potassium ion efflux triggers JNK kinase to phosphorylate NEK7, which regulates NLRP3 oligomerization and activation (47, 48). 3HP significantly downregulates NEK7 expression, further substantiating its pharmacological inhibition on NLRP3. Interestingly, we also found that 3HP markedly reduced the level of caspase-8. Previous studies have confirmed that in the presence of caspase-1, caspase-8 positively regulates the NLRP3/caspase-1 signaling cascade, which drives IL-1β production and cellular pyroptosis (49) and plays a crucial part in TLR4-mediated activation of the NLRP1/NLRP3 inflammasome (50). In light of these findings, our study confirmed the significant pharmacological inhibitory effect of 3HP on the LPS-stimulated NLRP3 inflammasome activation-mediated inflammatory cascade response. Additionally, to further evaluate the effect of 3HP on cellular pyroptosis, we examined the GSDMD protein family, which is closely associated with pyroptosis. In fact, the activation of the NLRP3 inflammasome induces specific cleavage of the GSDMD-N structural domain at the amino-terminal end of GSDMD by inflammatory caspase-1, resulting in pore formation at the cell membrane and initiating the release of inflammatory cytokines and cellular pyroptosis (51). In the current study, 3HP significantly inhibited the expression of Caspase-1 and Cleaved Caspase-1 p10, thus hindering the formation of pores in the membrane by the N-terminal structural domain of GSDMD and decreasing the pro-inflammatory cytokines, such as IL1β and IL18, from crossing the membrane pores to trigger the inflammatory cascade response, thereby inhibiting the cellular pyroptosis. MCC950, a potent and specific NLRP3 antagonist, directly targets the ATP hydrolysis motif of NLRP3 to inhibit the inflammasome (52). To further evaluate the protective effect of 3HP against NLRP3 activation, we conducted an *in vitro* experimental assay and found that MCC950 significantly enhanced the pharmacological inhibition of NLRP3 by 3HP. However, whether 3HP directly targets NLRP3 for pharmacological effects similar to MCC950 needs to be confirmed through more in-depth studies.

Considering TLR4 as a crucial upstream molecule for NF-κB/NLRP3 signaling activation, we investigated the interaction between 3HP and TLR4 through molecular docking and molecular dynamics simulations. Notably, 3HP exhibits stable binding with TLR4 within the pocket. Further analysis reveals that 3HP stabilizes interactions with TLR4 amino acid residues through electrostatic interactions, van der Waals forces, and hydrogen bonding, thereby exerting effective inhibitory effects. Meanwhile, pharmacologic inhibition results comparing the TLR4 selective inhibitor TAK-242 with 3HP indicate that 3HP may partially target TLR4 activation, thereby inhibiting downstream NF-κB/NLRP3 signaling and mitigating the onset and progression of ALI.

It is undeniable that the anti-inflammatory mechanism of dexamethasone, a glucocorticoid currently used for ALI treatment, may differ from that of 3HP. Therefore, in this study exploring the pharmacological mechanism by which 3HP improves ALI, we did not use dexamethasone or similar agents as positive controls. This represents a limitation in terms of the lack of comparative efficacy data between the two agents. Moreover, although RAW264.7 macrophages, as a cell line for *in vitro* classical models of inflammation, provide a useful research basis for understanding the pharmacological mechanisms of 3HP in alleviating ALI in this study. However, considering its inherent limitations, to better understand the molecular pharmacological mechanism of 3HP, we plan to use bone marrow-derived macrophages, THP-1 cells, and lung epithelial cells (MLE12) to explore the communication between macrophages and lung epithelial cells more deeply in our future work. We also aim to reveal cell-cell interactions in lung tissues through single-cell sequencing analyses, with the goal of providing additional experimental insights. Meanwhile, with ongoing innovation and advances in modern molecular pharmacology tools, such as activity-based protein profiling (ABPP), a technique that uses highly selective active-site targeted chemical probes to label and monitor the state of proteins (53), will help reveal the direct targeting molecules of 3HP to alleviate ALI and provide a new basis for clinical ALI treatment. While 3HP exhibits higher lipid solubility than puerarin (33), the oral bioavailability of isoflavones is generally limited by poor gastrointestinal absorption and first-pass metabolism (54).Our study utilized oral gavage, which is effective in mice but may require optimization for human application.We currently lack data on 3HP’s lung tissue concentration, a critical parameter for ALI treatment, as natural products often suffer from low tissue penetration and 3HP’s ability to reach the lung at therapeutic concentrations awaits verification; To address these challenges, future research can focus on the following directions: developing targeted delivery systems to improve 3HP’s oral bioavailability and lung tissue accumulation (55). Conducting *in vivo* pharmacokinetic analyses to clarify its absorption, distribution, metabolism, and excretion profiles, particularly lung tissue concentration-time curves; evaluating 3HP’s efficacy in large animal ALI models to more closely mimic human physiology; and exploring the synergistic effects of 3HP with low-dose dexamethasone to reduce adverse effects while enhancing therapeutic efficacy. Overall, our findings highlight the potent anti-inflammatory effects of 3HP and preliminarily reveal its significant inhibition of TLR4 activation-mediated NF-κB p65/NLRP3/GSDMD signaling in mitigating the onset and progression of ALI.

## 5 Conclusion

**Figure 9:**
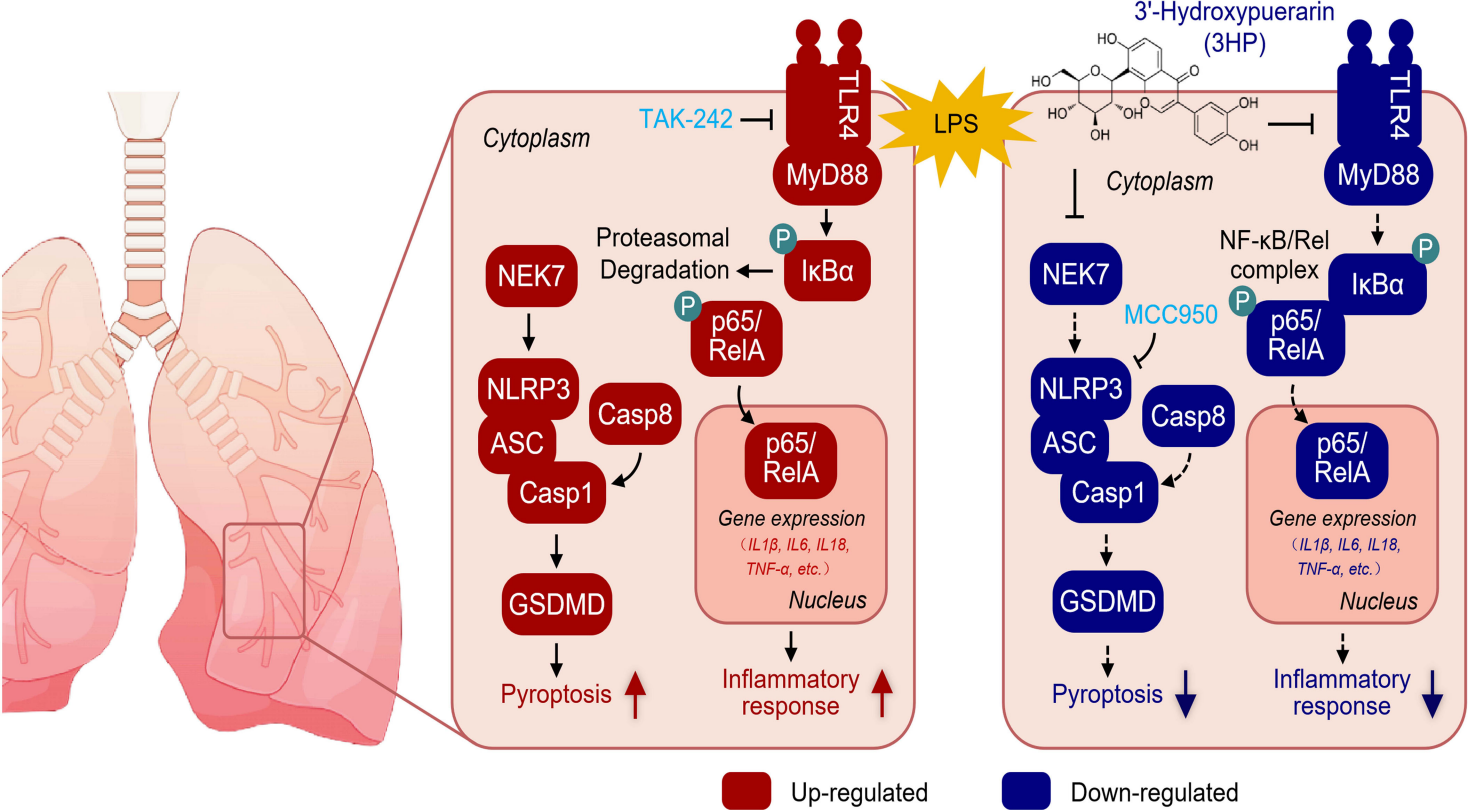
Pharmacological mechanism of 3HP in alleviating LPS-induced ALI in the current study.

Taken together, our results demonstrated that 3HP mitigated LPS-induced ALI by inhibiting TLR4 activation-mediated NF-κB p65/NLRP3/GSDMD signaling (**Figure 9**). These findings provide scientific evidence for the clinical treatment of ALI and present new insights into the pharmacological role of 3HP in mitigating acute lung inflammatory diseases.

## Data Availability

The original contributions presented in the study are included in the article/**Supplementary Material**. Further inquiries can be directed to the corresponding authors.

## References

[1] Johnson ER Matthay MA . Acute lung injury: epidemiology, pathogenesis, and treatment. J Aerosol Med Pulm Drug Delivery. (2010) 23:243–52. doi: 10.1089/jamp.2009.0775. PMID: 20073554 PMC3133560

[2] Mokrá D . Acute lung injury - from pathophysiology to treatment. Physiol Res. (2020) 69:S353–66. doi: 10.33549/physiolres.934602 PMC8603709 33464919

[3] Bos LDJ Ware LB . Acute respiratory distress syndrome: causes, pathophysiology, and phenotypes. Lancet. (2022) 400:1145–56. doi: 10.1016/s0140-6736(22)01485-4. PMID: 36070787

[4] Levitt JE Calfee CS Goldstein BA Vojnik R Matthay MA . Early acute lung injury: criteria for identifying lung injury prior to the need for positive pressure ventilation. Crit Care Med. (2013) 41:1929–37. doi: 10.1097/ccm.0b013e31828a3d99. PMID: 23782966 PMC3748809

[5] Hayes M Curley G Ansari B Laffey JG . Clinical review: Stem cell therapies for acute lung injury/acute respiratory distress syndrome - hope or hype? Crit Care. (2012) 16:205. doi: 10.1186/cc10570. PMID: 22424108 PMC3681334

[6] Herold S Mayer K Lohmeyer J . Acute lung injury: how macrophages orchestrate resolution of inflammation and tissue repair. Front Immunol. (2011) 2:65. doi: 10.3389/fimmu.2011.00065. PMID: 22566854 PMC3342347

[7] Cheng PY Li SY Chen HY . Macrophages in lung injury, repair, and fibrosis. Cells. (2021) 10:436. doi: 10.3390/cells10020436. PMID: 33670759 PMC7923175

[8] Kolomaznik M Nova Z Calkovska A . Pulmonary surfactant and bacterial lipopolysaccharide: the interaction and its functional consequences. Physiol Res. (2017) 66:S147–57. doi: 10.33549/physiolres.933672. PMID: 28937231

[9] Domscheit H Hegeman MA Carvalho N Spieth PM . Molecular dynamics of lipopolysaccharide-induced lung injury in rodents. Front Physiol. (2020) 11:36. doi: 10.3389/fphys.2020.00036. PMID: 32116752 PMC7012903

[10] Welbourn CR Young Y . Endotoxin, septic shock and acute lung injury: neutrophils, macrophages and inflammatory mediators. Br J Surg. (1992) 79:998–1003. doi: 10.1002/bjs.1800791006. PMID: 1422741

[11] Liu T Zhang LY Joo D Sun SC . NF-κB signaling in inflammation. Signal Transduct Target Ther. (2017) 2:17023. doi: 10.1038/sigtrans.2017.23. PMID: 29158945 PMC5661633

[12] Xu ZB Wang KY Hu HY Hu YJ Huang JW Luo ZH . Sinensetin attenuates LPS-induced acute pulmonary inflammation in mice and RAW264.7 cells by modulating NF-κB p65-mediated immune resistance and STAT3-mediated tissue resilience. Int Immunopharmacol. (2025) 148:114101. doi: 10.1016/j.intimp.2025.114101. PMID: 39827664

[13] Schroder K Sagulenko V Zamoshnikova A Richards AA Cridland JA Irvine KM . Acute lipopolysaccharide priming boosts inflammasome activation independently of inflammasome sensor induction. Immunobiology. (2012) 217:1325–9. doi: 10.1016/j.imbio.2012.07.020. PMID: 22898390

[14] Xu ZB Hu HY Wang KY Zhou ZY He XQ Huang XA . Sinensetin, a polymethoxyflavone from citrus fruits, ameliorates LPS-induced acute lung injury by suppressing Txnip/NLRP3/Caspase-1/GSDMD signaling-mediated inflammatory responses and pyroptosis. Food Funct. (2024) 15:7592–604. doi: 10.1039/d4fo01704h. PMID: 38938065

[15] Swanson KV Deng M Ting JPY . The NLRP3 inflammasome: molecular activation and regulation to therapeutics. Nat Rev Immunol. (2019) 19:477–89. doi: 10.1038/s41577-019-0165-0. PMID: 31036962 PMC7807242

[16] Ghayur T Banerjee S Hugunin M Butler D Herzog L Carter A . Caspase-1 processes IFN-gamma-inducing factor and regulates LPS-induced IFN-gamma production. Nature. (1997) 386:619–23. doi: 10.1038/386619a0. PMID: 9121587

[17] Guo HT Callaway JB Ting JP . Inflammasomes: mechanism of action, role in disease, and therapeutics. Nat Med. (2015) 21:677–87. doi: 10.1038/nm.3893. PMID: 26121197 PMC4519035

[18] Grailer JJ Canning BA Kalbitz M Haggadone MD Dhond RM Andjelkovic AV . Critical role for the NLRP3 inflammasome during acute lung injury. J Immunol. (2014) 192:5974–83. doi: 10.4049/jimmunol.1400368. PMID: 24795455 PMC4061751

[19] Gu WY Zeng Q Wang X Jasem H Ma L . Acute lung injury and the NLRP3 inflammasome. J Inflammation Res. (2024) 17:3801–13. doi: 10.2147/jir.s464838. PMID: 38887753 PMC11182363

[20] Tang JL Liu BY Ma KW . Traditional Chinese medicine. Lancet. (2008) 372:1938–40. doi: 10.1016/s0140-6736(08)61354-9. PMID: 18930523

[21] Wang Y Wang YC Ma J Li YN Cao L Zhu TX . YuPingFengSan ameliorates LPS-induced acute lung injury and gut barrier dysfunction in mice. J Ethnopharmacol. (2023) 312:116452. doi: 10.1016/j.jep.2023.116452. PMID: 37019161

[22] Liu YM Wang XJ Chen YL Zhou LM Wang YN Li LN . Pharmacological mechanisms of traditional Chinese medicine against acute lung injury: From active ingredients to herbal formulae. Phytomedicine. (2024) 135:155562. doi: 10.1016/j.phymed.2024.155562. PMID: 39536423

[23] Chang JS Wang KC Shieh DE Hsu FF Chiang LC . Ge-Gen-Tang has anti-viral activity against human respiratory syncytial virus in human respiratory tract cell lines. J Ethnopharmacol. (2012) 139:305–10. doi: 10.1016/j.jep.2011.11.018. PMID: 22120014

[24] Chen C Li XJ Kano Y Yuan D Qu JJ . Oriental traditional herbal medicine--Puerariae Flos: A systematic review. J Ethnopharmacol. (2023) 306:116089. doi: 10.1016/j.jep.2022.116089. PMID: 36621660

[25] Shree P Mishra P Kumar P Pandey H Giri R Chaube R . In silico screening of Pueraria tuberosa (PTY-2) for targeting COVID-19 by countering dual targets Mpro and TMPRSS2. J Biomol Struct Dyn. (2022) 40:11611–24. doi: 10.1080/07391102.2021.1965029 34424815

[26] Du H Shao MJ Xu SC Yang Q Xu JQ Ke H . Integrating metabolomics and network pharmacology analysis to explore mechanism of Pueraria lobata against pulmonary fibrosis: Involvement of arginine metabolism pathway. J Ethnopharmacol. (2024) 332:118346. doi: 10.1016/j.jep.2024.118346. PMID: 38782311

[27] Lu Y Xu J Tang R Zeng PY Li ZY You JC . Edible pueraria lobata-derived exosome-like nanovesicles ameliorate dextran sulfate sodium-induced colitis associated lung inflammation through modulating macrophage polarization. BioMed Pharmacother. (2024) 170:116098. doi: 10.1016/j.biopha.2023.116098. PMID: 38154276

[28] Dong HY Zhao Y Teng H Jiang T Yue YH Zhang S . Pueraria lobata antioxidant extract ameliorates non-alcoholic fatty liver by altering hepatic fat accumulation and oxidative stress. J Ethnopharmacol. (2024) 333:118468. doi: 10.1016/j.jep.2024.118468. PMID: 38906339

[29] Kaufman PB Duke JA Brielmann H Boik J Hoyt JE . A comparative survey of leguminous plants as sources of the isoflavones, genistein and daidzein: implications for human nutrition and health. J Altern Complement Med. (1997) 3:7–12. doi: 10.1089/acm.1997.3.7. PMID: 9395689

[30] Prasain JK Jones K Kirk M Wilson L Smith-Johnson M Weaver C . Profiling and quantification of isoflavonoids in kudzu dietary supplements by high-performance liquid chromatography and electrospray ionization tandem mass spectrometry. J Agric Food Chem. (2003) 51:4213–8. doi: 10.1021/jf030174a. PMID: 12848487

[31] Wang XY Yan JJ Xu XH Duan CY Xie Z Su ZQ . Puerarin prevents LPS-induced acute lung injury via inhibiting inflammatory response. Microb Pathog. (2018) 118:170–6. doi: 10.1016/j.micpath.2018.03.033. PMID: 29571724

[32] Cai DS Zhao Y Yu F . Puerarin ameliorates acute lung injury by modulating NLRP3 inflammasome-induced pyroptosis. Cell Death Discov. (2022) 8:368. doi: 10.1038/s41420-022-01137-8. PMID: 35977927 PMC9385627

[33] Guo N Fang ZY Zang QC Yang YQ Nan TG Zhao YP . Spatially resolved metabolomics combined with bioactivity analyses to evaluate the pharmacological properties of two Radix Puerariae species. J Ethnopharmacol. (2023) 313:116546. doi: 10.1016/j.jep.2023.116546. PMID: 37121451

[34] Jin SE Son YK Min BS Jung HA Choi JS . Anti-inflammatory and antioxidant activities of constituents isolated from Pueraria lobata roots. Arch Pharm Res. (2012) 35:823–37. doi: 10.1007/s12272-012-0508-x. PMID: 22644850

[35] Xu ZB Li JY Zhou KL Wang KY Hu HY Hu YJ . Exocarpium Citri Grandis ameliorates LPS-induced acute lung injury by suppressing inflammation, NLRP3 inflammasome, and ferroptosis. J Ethnopharmacol. (2024) 329:118162. doi: 10.1016/j.jep.2024.118162 38588989

[36] Fan E Brodie D Slutsky AS . Acute respiratory distress syndrome: advances in diagnosis and treatment. JAMA. (2018) 319:698–710. doi: 10.1001/jama.2018.5932. PMID: 29466596

[37] Millar MW Fazal F Rahman A . Therapeutic targeting of NF-κB in acute lung injury: a double-edged sword. Cells. (2022) 11:3317. doi: 10.3390/cells11203317. PMID: 36291185 PMC9601210

[38] Malainou C Abdin SM Lachmann N Matt U Herold S . Alveolar macrophages in tissue homeostasis, inflammation, and infection: evolving concepts of therapeutic targeting. J Clin Invest. (2023) 133:e170501. doi: 10.1172/jci170501. PMID: 37781922 PMC10541196

[39] Lazarus HM Fox J Burke JP Lloyd JF Snow GL Mehta RR . Trauma patient hospital-associated infections: risks and outcomes. J Trauma. (2005) 59:188–94. doi: 10.1097/01.ta.0000171535.75484.df. PMID: 16096562

[40] Zhang JG Guo YM Mak M Tao ZM . Translational medicine for acute lung injury. J Transl Med. (2024) 22:25. doi: 10.1186/s12967-023-04828-7. PMID: 38183140 PMC10768317

[41] Xu J Yang Y . Traditional Chinese medicine in the Chinese health care system. Health Policy. (2009) 90:133–9. doi: 10.1016/j.healthpol.2008.09.003. PMID: 18947898 PMC7114631

[42] Latz E Xiao TS Stutz A . Activation and regulation of the inflammasomes. Nat Rev Immunol. (2013) 13:397–411. doi: 10.1038/nri3452. PMID: 23702978 PMC3807999

[43] Franchi L Muñoz-Planillo R Núñez G . Sensing and reacting to microbes through the inflammasomes. Nat Immunol. (2012) 13:325–32. doi: 10.1038/ni.2231. PMID: 22430785 PMC3449002

[44] Liu YM Meng JY Liu J Zhang F An C Yang J . NLRP3 inflammasome in acute lung injury: cumulative evidence for traditional Chinese medicine and natural products. Chin Herb Med. (2025) 17:703–19. doi: 10.1016/j.chmed.2025.06.002. PMID: 41399792 PMC12702441

[45] Wang YC Liu QX Zheng Q Liu T Xu XE Liu XH . Dihydromyricetin alleviates sepsis-induced acute lung injury through inhibiting NLRP3 inflammasome-dependent pyroptosis in mice model. Inflammation. (2019) 42:1301–10. doi: 10.1007/s10753-019-00990-7. PMID: 30887396

[46] Sharif H Wang L Wang WL Magupalli VG Andreeva L Qiao Q . Structural mechanism for NEK7-licensed activation of NLRP3 inflammasome. Nature. (2019) 570:338–43. doi: 10.1038/s41586-019-1295-z. PMID: 31189953 PMC6774351

[47] He Y Zeng MY Yang DH Motro B Núñez G . NEK7 is an essential mediator of NLRP3 activation downstream of potassium efflux. Nature. (2016) 530:354–7. doi: 10.4049/jimmunol.196.supp.62.3. PMID: 26814970 PMC4810788

[48] Xu J Zhang LZ Duan YH Sun FY Odeh N He Y . NEK7 phosphorylation amplifies NLRP3 inflammasome activation downstream of potassium efflux and gasdermin D. Sci Immunol. (2025) 10:eadl2993. doi: 10.4049/jimmunol.212.supp.0180.4791. PMID: 39752537 PMC12020992

[49] Antonopoulos C Russo HM El Sanadi C Martin BN Li X Kaiser WJ . Caspase-8 as an effector and regulator of NLRP3 inflammasome signaling. J Biol Chem. (2015) 290:20167–84. doi: 10.1074/jbc.m115.652321. PMID: 26100631 PMC4536427

[50] Chi W Li F Chen HR Wang YD Zhu YT Yang XJ . Caspase-8 promotes NLRP1/NLRP3 inflammasome activation and IL-1β production in acute glaucoma. Proc Natl Acad Sci USA. (2014) 111:11181–6. doi: 10.1073/pnas.1402819111. PMID: 25024200 PMC4121847

[51] Shi JJ Zhao Y Wang K Shi XY Wang Y Huang HW . Cleavage of GSDMD by inflammatory caspases determines pyroptotic cell death. Nature. (2015) 526:660–5. doi: 10.1038/nature15514. PMID: 26375003

[52] Coll RC Hill JR Day CJ Zamoshnikova A Boucher D Massey NL . MCC950 directly targets the NLRP3 ATP-hydrolysis motif for inflammasome inhibition. Nat Chem Biol. (2019) 15:556–9. doi: 10.1038/s41589-019-0277-7. PMID: 31086327

[53] Fang H Peng B Ong SY Wu Q Li L Yao SQ . Recent advances in activity-based probes (ABPs) and affinity-based probes (AfBPs) for profiling of enzymes. Chem Sci. (2021) 12:8288–310. doi: 10.1039/d1sc01359a. PMID: 34221311 PMC8221178

[54] De Andrade CM Bianchini FJ Rey FM Fonseca MJ Toloi MR . Effects of an aglycone-rich biotransformed soybean extract in human endothelial cells. Climacteric. (2015) 18:651–5. doi: 10.3109/13697137.2014.981519. PMID: 25530207

[55] Andra VVSNL Pammi SVN Bhatraju LVKP Ruddaraju LK . A comprehensive review on novel liposomal methodologies, commercial formulations, clinical trials and patents. Bionanoscience. (2022) 12:274–91. doi: 10.1007/s12668-022-00941-x. PMID: 35096502 PMC8790012

