# Supplementary material for: 3’-hydroxypuerarin mitigates LPS-induced acute lung injury by inhibiting TLR4 activation-mediated NF-κB p65/NLRP3/GSDMD signaling
Source: Front Immunol. 2026 Mar 31;17:1701778. doi: 10.3389/fimmu.2026.1701778 (PMC13076151; doi:10.3389/fimmu.2026.1701778)
Supplement: Supplementary file 1 [file Table1.docx]

**Supplementary Material**

**Supplementary Table 1**. Primers used in qPCR analysis.

| **Gene** | **Forward Primer (5' → 3')** | **Reward Primer (5' → 3')** |
| --- | --- | --- |
| *IL1α* | CGAAGACTACAGTTCTGCCATT | GACGTTTCAGAGGTTCTCAGAG |
| *IL1β* | CCGTGGACCTTCCAGGATGA | GGGAACGTCACACACCAGCA |
| *IL6* | TAGTCCTTCCTACCCCAATTTCC | TTGGTCCTTAGCCACTCCTTC |
| *TNF-α* | AGCCCCCAGTCTGTATCCTT | CTCCCTTTGCAGAACTCAGG |
| *Ccl2* | TTAAAAACCTGGATCGGAACCAA | GCATTAGCTTCAGATTTACGGGT |
| *Ccl3* | TTCTCTGTACCATGACACTCTGC | CGTGGAATCTTCCGGCTGTAG |
| *Ccl4* | TTCCTGCTGTTTCTCTTACACCT | CTGTCTGCCTCTTTTGGTCAG |
| *Ccl5* | TCGAGTGACAAACACGACTGC | GCTGCTTTGCCTACCTCTCC |
| *Ccl7* | GCTGCTTTCAGCATCCAAGTG | CCAGGGACACCGACTACTG |
| *Cxcl1* | CTGGGATTCACCTCAAGAACATC | CAGGGTCAAGGCAAGCCTC |
| *Cxcl2* | CCAACCACCAGGCTACAGG | GCGTCACACTCAAGCTCTG |
| *Cxcl9* | TCCTTTTGGGCATCATCTTCC | TTTGTAGTGGATCGTGCCTCG |
| *Cxcl10* | CCAAGTGCTGCCGTCATTTTC | GGCTCGCAGGGATGATTTCAA |
| *F4/80* | CCCCAGTGTCCTTACAGAGTG | GTGCCCAGAGTGGATGTCT |
| *IFN-γ* | CTTCAGCAACAGCAAGGC | CGAATCAGCAGCGACTCC |
| *NLRP3* | TGTGAGAAGCAGGTTCTACTCT | TGTAGCGACTGTTGAGGTCCA |
| *ASC* | CTTGTCAGGGGATGAACTCAAAA | GCCATACGACTCCAGATAGTAGC |
| *IL18* | GACTCTTGCGTCAACTTCAAGG | CAGGCTGTCTTTTGTCAACGA |
| *β-actin* | GGCTGTATTCCCCTCCATCG | CCAGTTGGTAACAATGCCATGT |
