# Supplementary material for: 3’-hydroxypuerarin mitigates LPS-induced acute lung injury by inhibiting TLR4 activation-mediated NF-κB p65/NLRP3/GSDMD signaling
Source: Front Immunol. 2026 Mar 31;17:1701778. doi: 10.3389/fimmu.2026.1701778 (PMC13076151; doi:10.3389/fimmu.2026.1701778)
Supplement: Supplementary file 2 [file Table2.docx]

**Supplementary Table 2**. The binding energy by MMPBSA (kcal/mol)

| Type | TLR4-3HP |
| --- | --- |
| *E_VDW_* | -22.46 ± 0.87 |
| *E_ELE_* | -85.01 ± -5.4 |
| *E_GB_* | 77.13 ± -1.65 |
| *E_SA_* | -3.14 ± -0.01 |
| *G_Binding energy_* | -33.48 ± -5.71 |

*E_VDW_*: van der Waals energy

*E_ELE_*: eletrostatic energy

*E_GB_*: polar contribution to solvation

*E_SA_*: non-polar contribution to solvation
